# Serological responses to human virome define clinical outcomes of Italian patients infected with SARS-CoV-2

**DOI:** 10.7150/ijbs.78002

**Published:** 2022-09-01

**Authors:** Limin Wang, Julián Candia, Lichun Ma, Yongmei Zhao, Luisa Imberti, Alessandra Sottini, Eugenia Quiros-Roldan, Kerry Dobbs, Peter D. Burbelo, Jeffrey I. Cohen, Ottavia M. Delmonte, Marshonna Forgues, Hui Liu, Helen F. Matthews, Elana Shaw, Michael A. Stack, Sarah E. Weber, Yu Zhang, Andrea Lisco, Irini Sereti, Helen C. Su, Luigi D. Notarangelo, Xin Wei Wang

**Affiliations:** 1 Laboratory of Human Carcinogenesis, Center for Cancer Research, National Cancer Institute, NIH, Bethesda, Maryland 20892; 2 CCR-SF Bioinformatics Group, Advanced Biomedical and Computational Sciences, Frederick National Laboratory for Cancer Research, 8560 Progress Drive, Frederick, Maryland 21701; 3 CREA Laboratory, Diagnostic Department, ASST Spedali Civili di Brescia, Brescia, Italy; 4 Department of Infectious and Tropical Diseases, University of Brescia and ASST Spedali Civili, Brescia, Italy; 5 Laboratory of Clinical Immunology and Microbiology, National Institute of Allergy and Infectious Diseases, NIH, Bethesda, Maryland 20892; 6 National Institute of Dental and Craniofacial Research, NIH, Bethesda, Maryland 20892; 7 Laboratory of Infectious Diseases, National Institute of Allergy and Infectious Diseases, NIH, Bethesda, Maryland 20892; 8 Laboratory of Immunology, National Institute of Allergy and Infectious Diseases, NIH, Bethesda, Maryland 20892; 9 Section of Molecular Development of the Immune System, National Institute of Allergy and Infectious Diseases, NIH, Bethesda, Maryland 20892; 10 Laboratory of Immunoregulation, National Institute of Allergy and Infectious Diseases, NIH, Bethesda, Maryland 20892; 11 Liver Cancer Program, Center for Cancer Research, National Cancer Institute, NIH, Bethesda, Maryland 20892; 12 These authors contributed equally; 13 Lead Contact

## Abstract

Severe acute respiratory syndrome coronavirus 2 (SARS-CoV-2) is responsible for the pandemic respiratory infectious disease COVID-19. However, clinical manifestations and outcomes differ significantly among COVID-19 patients, ranging from asymptomatic to extremely severe, and it remains unclear what drives these disparities. Here, we studied 159 sequentially enrolled hospitalized patients with COVID-19-associated pneumonia from Brescia, Italy using the VirScan phage-display method to characterize circulating antibodies binding to 96,179 viral peptides encoded by 1,276 strains of human viruses. SARS-CoV-2 infection was associated with a marked increase in immune antibody repertoires against many known pathogenic and non-pathogenic human viruses. This antiviral antibody response was linked to longitudinal trajectories of disease severity and was further confirmed in additional 125 COVID-19 patients from the same geographical region in Northern Italy. By applying a machine-learning-based strategy, a viral exposure signature predictive of COVID-19-related disease severity linked to patient survival was developed and validated. These results provide a basis for understanding the role of memory B-cell repertoire to viral epitopes in COVID-19-related symptoms and suggest that a unique anti-viral antibody repertoire signature may be useful to define COVID-19 clinical severity.

## Introduction

Coronavirus disease-2019 (COVID-19) is a severe acute respiratory disease caused by the infection of SARS-CoV-2, which belongs to a group of virus es with a positive-sense, single-stranded RNA genome, similar to other known β-coronaviruses including SARS-CoV (also known as SARS-CoV-1), MERS-CoV, and other seasonal and less pathogenic coronaviruses (e.g., HKU and OC43) 1-7.  SARS-CoV-2, discovered in January 2020, is directly responsible for more than 115 million confirmed cases and 2.56 million deaths globally, as of March 4, 2021. SARS-CoV-2 infection is associated with a wide spectrum of clinical manifestations that range from an asymptomatic infection to lower respiratory tract involvement and multifocal interstitial pneumonia, in some cases progressing to severe hypoxic respiratory failure with acute respiratory distress syndrome (ARDS), multiorgan failure, and death 8. The clinical course and animal models of COVID-19 disease suggest a biphasic trajectory initially dominated by innate and early adaptive immune responses that can result in viral clearance and clinical resolution, but in cases of ineffective early responses, by a persistent and dysregulated inflammatory response associated with high morbidity and mortality. It is unclear, however, why some patients are asymptomatic while others develop severe symptoms. Among hospitalized patients, older patients 9, 10 and cancer patients are more likely to develop severe disease 11-13. As COVID-19-related severe clinical presentations are associated with persistent viral shedding and dysregulated inflammatory response, it is plausible that ineffective host immune responses may contribute to determine the severity of clinical manifestations 14.

Viruses may affect human health by altering host immunity, contributing to the pathogenesis of inflammatory disorders such as autoimmune disease and cancer 15-17. Various human viruses may interact with one another in the host and may alter a host's response to new infections and thereby affect disease severity. It is known that viruses that persist or are cleared in the host may leave unique molecular footprints, known as viral epitope-specific reactive antibodies, that can affect host susceptibility to other infections, which may be a surrogate of disease severity and progression. For example, prior infection of human herpesvirus 5 (CMV) could improve immune response to influenza 18. The recent identification of cross-reactive T cells to SARS-CoV-2 in unexposed individuals may provide a hint about a possible impact of past exposure to seasonal coronaviruses on COVID-19-related outcomes 19, 20. Thus, the pattern and type of serological responses to human viruses represent a unique immunological, host-specific signature that provides insight into the history of viral exposures, host immunity of each individual, and disease onset.

We have recently employed a synthetic virome technology, VirScan, to detect the exposure history to most known human viruses 21. VirScan implements a phage display library that covers 96,179 viral peptides that are each 56 residues in length tiling the protein sequences with 28 residue overlaps, which corresponds to 206 viral species and 1,276 human viral strains 22. Using this technology, we developed a viral exposure signature (VES) that could discriminate liver cancer patients from at-risk or healthy individuals and validated the results in a longitudinal cohort with at-risk patients who had long-term follow-up for liver cancer development 21. Remarkably, VES could predict cancer among at-risk patients prior to a clinical diagnosis and appeared clinically applicable for liver cancer surveillance. In the present study, we show how VirScan may provide a sensitive approach to identify immunological footprints predictive of different clinical outcomes of SARS-CoV-2 infection. With that aim, we performed serological profiles of 284 hospitalized patients with pneumonia who were suspected to be infected with SARS-CoV-2, as part of the NIAID-NCI COVID-19 Consortium studies.

## Results

**Figure 1:**
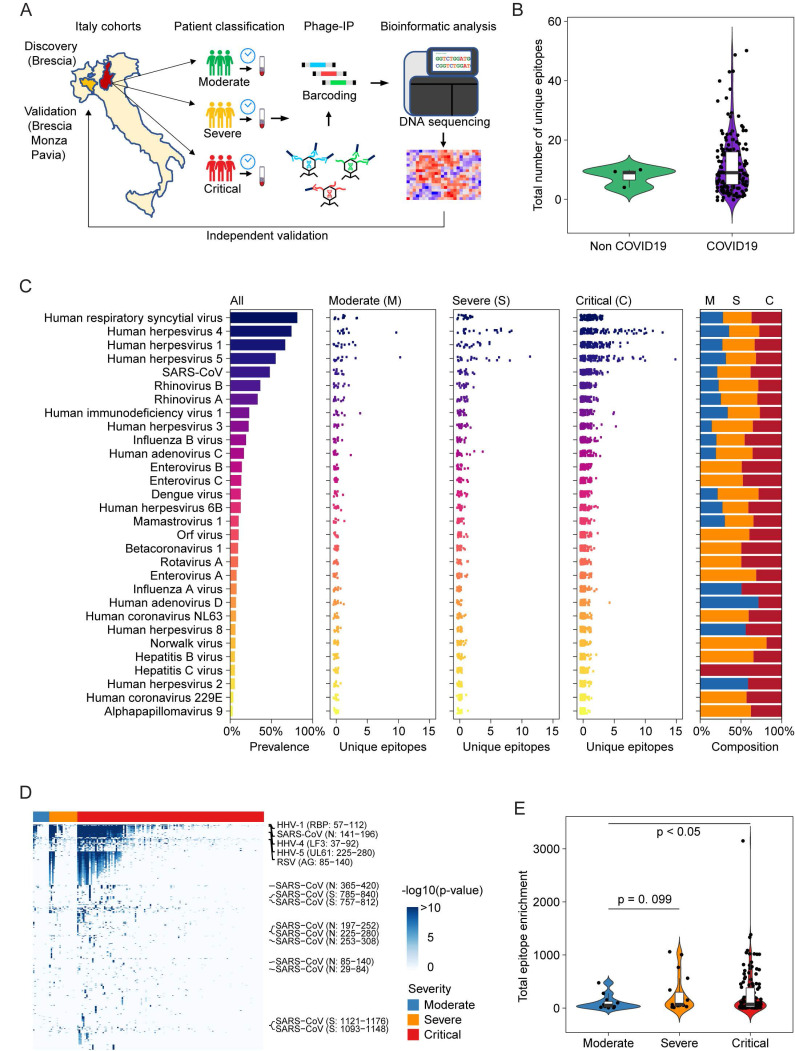
**Overview of viral exposure across Italian COVID-19 cohorts.** (**A**) Discovery and validation workflow of our VirScan study. (**B**) Total number of unique epitopes in non-COVID-19 and COVID-19 cases in the Brescia discovery cohort. (**C**) Viral prevalence (left), number of unique epitopes of moderate, severe, and critical groups of patients (3 center panels) and composition of prevalence across patient groups (right) for 156 COVID-19 cases in the Brescia discovery cohort. (**D**) Antibody reactivity of all epitopes detected in at least two cases. Rows represent epitopes and columns represent COVID-19 cases from the Brescia discovery cohort, where -log10 (p-value) was used to quantify peptide enrichment. (**E**) Log-transformed total enrichment across all epitopes in the moderate, severe, and critical groups from the Brescia discovery cohort. For each violin plot, the embedded box spans the interquartile range around the median (thick horizontal line), whereas the contour denotes the kernel density estimate of the distribution. Box plots represent 25^th^ to 75^th^ percentiles and whiskers extend to 10^th^ and 90^th^ percentiles. P-values were determined with Student's t-test.

Among 159 sequentially enrolled hospitalized patients with pneumonia in Brescia, Italy in the discovery cohort, 156 patients tested positive and 3 patients negative for the presence of SARS-CoV-2 infection by qRT-PCR and/or serology tests. In this cohort, 73% of patients were male and the median age was 60 years. Twelve patients were classified as moderate, 19 as severe, and 128 as critical (Table 1). Among the latter, 30 patients died during hospitalization. We used version 2 of the VirScan phage library to profile this cohort (Figure 1A), which yielded on average 2 million single-end reads per serum sample with mean mapped reads of 91%, comparable to the Maryland (NCI-UMD) cohort recently described 21. Using replicates for setting up the reproducibility threshold with built-in blank replica for each plate as background controls, we obtained 16,536 significantly enriched viral epitopes. We detected antibodies reactive to a median of six species of virus per sample, slightly lower than the median of seven species of virus per sample found among healthy individuals from the Baltimore area in the United States 21. Interestingly, while we found antibodies reactive to a median of 9 viral epitopes per sample in the Brescia cohort, there was an elevation of total reactive antibodies to known viruses in COVID-19 patients compared to non-COVID-19 patients, although it did not reach statistical significance (Figure 1B). The prevalence of antibodies reactive to the most common viruses, such as human respiratory syncytial virus (HRSV), related to respiratory tract infections, human herpesvirus 1 (HHV1), related to oral lesions and encephalitis, human herpesvirus 4 (EBV), related to infectious mononucleosis and human herpesvirus 5 (CMV), was comparable between Brescia COVID-19 patients (Figure 1C) and healthy individuals from Baltimore, as well as other populations 21, 22. Interestingly, we observed some variations of antibodies for viral compositions among moderate, severe, and critical patients with a tendency of an increasing level of antibodies reactive to unique viral epitopes corresponding to disease severity (Figure 1C), and this was confirmed by a quantitative analysis of the total amount of antibodies reactive to viral epitopes across clinical severity patient groups (Figure 1, D and E). There was a statistically significant increase in reactive antibodies with increasing severity in clinical presentations. Noticeably, SARS-CoV antibodies were detected in 27% of moderate patients but 53% of severe patients and 49% of critical patients (Figure 1C, Supplemental Table 1). The difference of viral antibody composition and levels of antibodies reactive to unique epitopes was comparatively small when stratified by age and sex (Supplemental Figure 1, A-D). Similar results were also observed in a validation cohort of 125 COVID-19 patients from three hospitals in the region of Lombardy, Northern Italy (Supplemental Figure 1E). Remarkably, the levels of viral antibodies were significantly elevated in patients in the acute phase of COVID-19 but declined in convalescent patients (Supplemental Figure 1E). Taken together, these results indicate that an individual's immune memory antibody repertoires due to a history of viral exposure showed a marked increase during acute SARS-CoV-2 infection.

**Figure 2:**
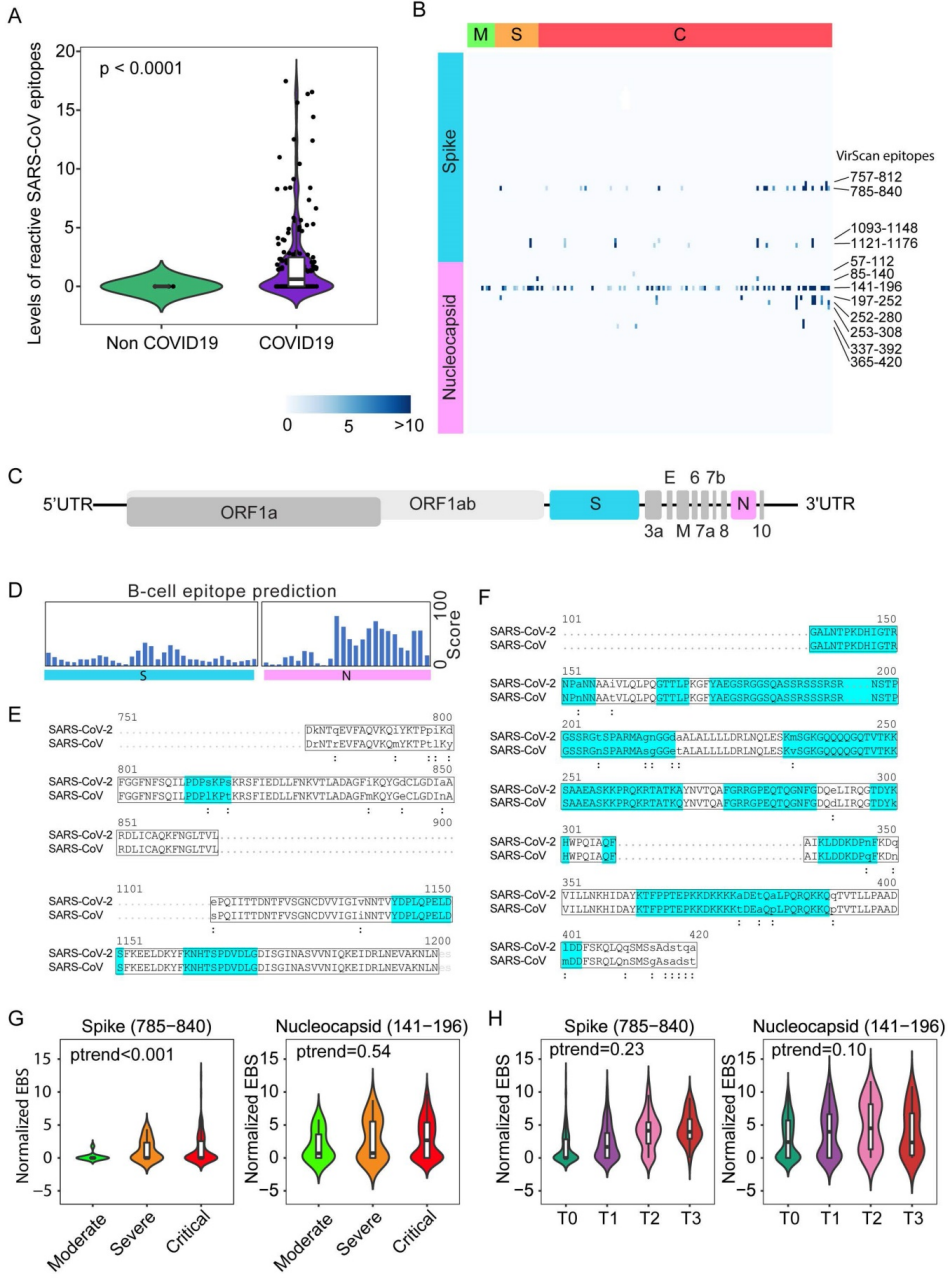
** SARS-CoV epitope reactivity in moderate, severe, and critical cases from the Brescia discovery cohort.** (**A**) Total reactivity of all SARS-CoV epitopes in non-COVID and COVID-19 cases. Log transformation was applied. (**B**) Antibody reactivity of 85 SARS-CoV epitopes. Each row represents the significant peptide tiling corresponding to spike protein (1-1255) and nucleocapsid protein (1-422). The color intensity of each cell corresponds to the scaled -log10(p value) measure of significance of enrichment for a peptide in a sample. (**C**) Organization of SARS-CoV-2 genome encoding various viral proteins. (**D**) B-cell epitope prediction score for spike and nucleocapsid based on the Immune Epitope Database and Analysis Resource (IEDB). (**E-F**) Sequence alignment of reactive peptides corresponding to spike (**E**) and nucleocapsid protein (**F**) of SARS-CoV and SARS-CoV-2. Only peptide sequences in the phage library are shown. Residues with perfect match are capitalized. Predicted epitopes by IEDB are highlighted. (**G**) Normalized EBS of spike (785-840, left) and nucleocapsid (141-196 right) proteins in moderate, severe, and critical patients. (**H**) Normalized EBS of spike (785-840, left) and nucleocapsid (141-196 right) proteins in patients at different time points. In violin plots, boxes span the interquartile range; lines within boxes represent the median; the width of violin plots indicates the kernel density of values. Box plots represent 25^th^ to 75^th^ percentiles and whiskers extend to 10^th^ and 90^th^ percentiles. P-values were determined with Student's t-test.

Nearly 50% of COVID-19 patients from Brescia had antibodies reactive to SARS-CoV peptides (Figure 1C, left panel). We also detected antibodies reactive to peptides corresponding to several common coronaviruses including NL63, 229E, HKU1 and OC43, but their prevalence was low (7%, 4%, 2% and 1%, respectively). The levels of SARS-CoV positive cases were unexpectedly high in Brescia patients, given the fact that reactive antibodies to SARS-CoV epitope sequences were hardly detectable in our previous VirScan study of 899 Baltimore patients and other populations, suggesting that the SARS-CoV prevalence is extremely low in the general population 21, 22. Consistently, SARS-CoV reactivity could not be detected in patients diagnosed with pneumonia that tested negative for SARS-CoV-2 while its level was high in COVID-19 patients, in both the discovery cohort (Figure 2A) and the validation cohort (Supplemental Figure 2A). Noticeably, SARS-CoV reactivity was undetectable in 6 convalescent patients in the validation cohort. We hypothesized that SARS-CoV peptides used in the phage library may detect SARS-CoV-2 cross-reactive antibodies. Upon close examination of the VirScan phage library, we found that it contained 80 epitope tiles corresponding to the SARS-CoV sequence, namely 44 epitopes for the spike protein, 15 epitopes for the nucleocapsid protein, and 21 epitopes for 3a, 3b, 7a, and 9b proteins. Among them, 4 peptides spanning the spike protein sequence and 8 peptides spanning the nucleocapsid protein showed strong reactive signals to COVID-19 positive patients (Figure 2B). Since SARS-CoV and SARS-CoV-2 share a similar viral structure (Figure 2C) with an overall 79% genetic similarity, we determined sequence similarities between SARS-CoV epitopes used in the library and the newly identified SARS-CoV-2 sequences.

We found that reactive SARS-CoV epitope sequences share a high homology to SARS-CoV-2, especially in the regions predicted to be strongly antigenic by the B-cell epitope prediction tool of the Immune Epitope Database (http://tools.iedb.org) (Figure 2, D-F). Thus, we concluded that VirScan's SARS-CoV peptides may be effectively used to detect SARS-CoV-2 antibodies. We measured the antibody epitope binding signal (EBS) to estimate the antibody titer for each epitope recently described 23. Similar to an overall increase in the immune memory antibody repertoire diversity to all known pathogenic viruses corresponding to clinical severity, we found a significant increase in levels of antibodies to the spike protein, but not to the nucleocapsid protein, with increased disease severity (Figure 2G). We also found an increasing trend in antibody production to the spike protein over time during hospitalization (Figure 2H). Consistently, a significant increase in antibodies to the spike protein was found in patients requiring intensive care unit (ICU) care compared with non-ICU patients (Supplemental Figure 2B). However, no statistical difference was observed for antibodies against the nucleocapsid protein (Supplemental Figure 2C). Moreover, levels of anti-Spike antibodies did not correlate with mortality (Supplemental Figure 2D). To further validate the above finding, we performed correlation analysis of antibody reactivity against spike and nucleocapsid proteins between VirScan and the luciferase immunoprecipitation system (LIPS) that employs an immunoassay to detect both linear and conformational antibodies against full-length nucleocapsid and spike proteins 24. We found a significant concordance between VirScan and LIPS (Supplemental Figure 2, E and F). In contrast, there was no correlation between the total amount of reactive antibodies detected by VirScan and the levels of circulating immunoglobulin (Supplemental Figure 2, G and H). However, consistent with the results of the discovery cohort, we found that the total amount of antibodies reactive to all viral epitopes was significantly elevated corresponding to the disease severity among patients in the validation cohort (Supplemental Figure 2I).

**Figure 3:**
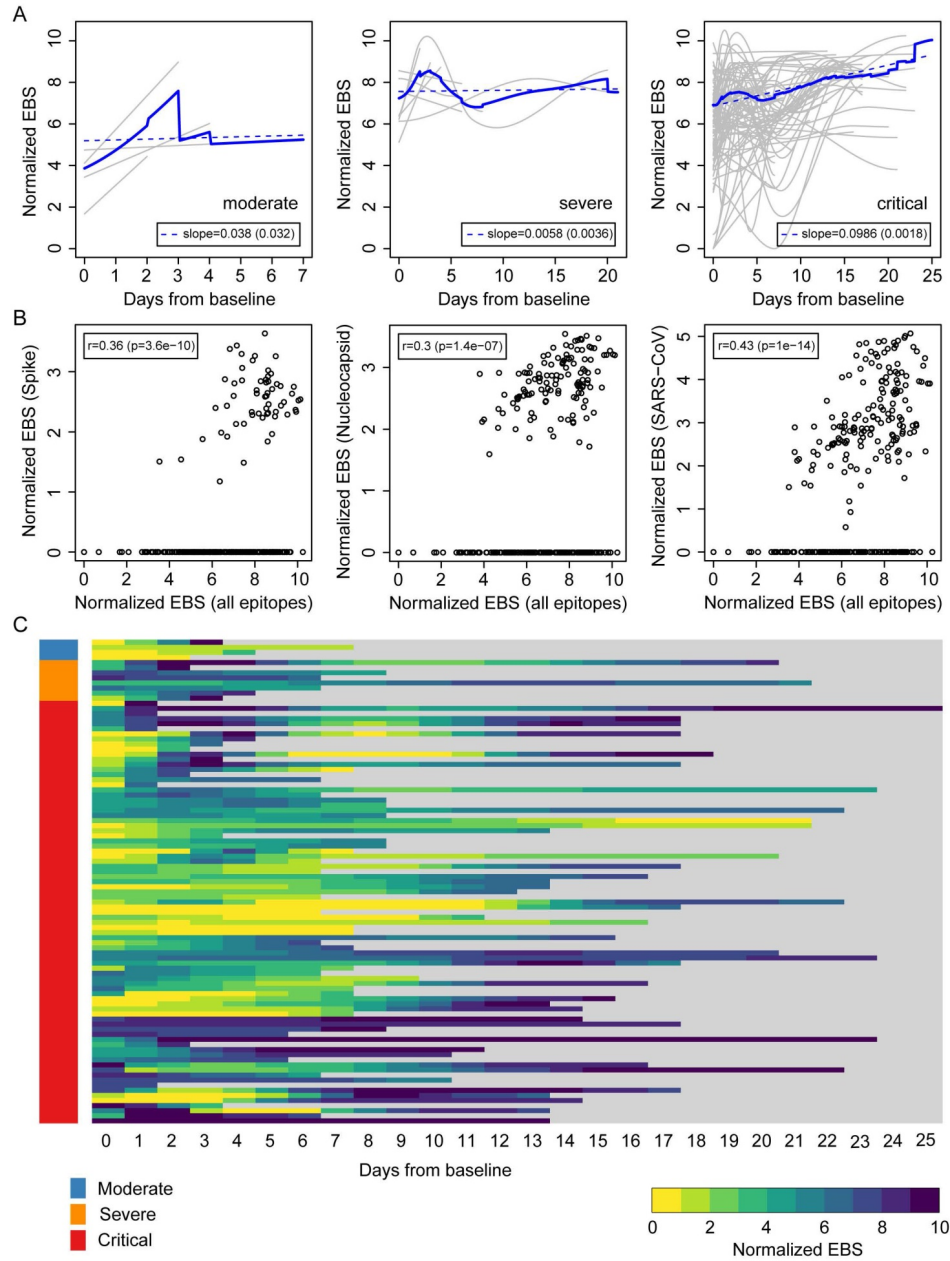
**Longitudinal progression of the normalized EBS across individuals from the Brescia discovery cohort.** (**A**) Individual trajectories over time for patients grouped by disease severity (gray lines), which were averaged (solid blue line) and fitted by linear regression (dashed blue line; slope and standard error shown in the legend). Baseline refers to the first sample obtained after admission to the hospital. (**B**) Normalized EBS in SARS-CoV spike, nucleocapsid, and all SARS-CoV reactive proteins compared against the normalized EBS across all VirScan epitopes. (**C**) Heatmap showing the longitudinal progression of individual patients integrated across all three patient groups by disease severity. P-values were determined with Student's t-test.

The above results suggest that an individual's immune memory antibody repertoires may be modulated upon acute infection by SARS-CoV-2. To further explore this hypothesis, we tracked the longitudinal progression of EBS for individual patients. Figure 3A shows the individual trajectories over time for COVID-19 patients grouped by disease severity (gray lines), which were generated from discrete timepoints using LOESS regression. The average trajectory for each group (solid blue line) was then fitted by linear regression (dashed blue line) to extract the slope's value and standard error (shown in the legend). While the rate of change of the moderate and severe groups is almost flat (i.e. the slope is consistent with zero after taking the standard error into account), the critical group shows a significant positive slope. Therefore, in addition to having an increased EBS at baseline, COVID-19 patients in the critical group display a characteristic overall increase across the entire antibody repertoire over time. Consistent with these observations, the EBS rate of change among ICU patients is observed to be significantly larger compared with that of non-ICU patients (Supplemental Figure 3A) and, analogously, the EBS slope among deceased patients appears significantly larger than that of survivors (Supplemental Figure 3B). Furthermore, similar analyses of sex and age effects on the humoral immune response of COVID-19 patients show that both the total epitope enrichment at baseline (Supplemental Figure 4, A and B) and the longitudinal EBS progression (Supplemental Figure 4, C-F) increase with age and are significantly higher among males compared with female patients. In agreement with well-established observations of older patients, preferentially male, being more susceptible to severe COVID-19 progression and death 9, 10, 25, our findings solidify the emerging picture of disease severity being robustly associated with an elevated, non-specific humoral immune activation. The EBS signal calculated across all epitopes (horizontal axis) correlated with the EBS signal from the 785-840 epitope in the spike protein, the 141-196 epitope in the nucleocapsid protein, and all available SARS-CoV peptide sequences, respectively (Figure 3B). While, as expected, restricting the EBS signal to one or a few epitopes yields noisier measurements, we nonetheless observe significant positive correlations with the overall EBS across the cohort. The heatmap in Figure 3C shows in greater detail the integrated longitudinal EBS progression from the three patient groups (indicated by the color bar on the left), which emphasizes the intriguing complexity and heterogeneity of the COVID-19 time course.

To determine if virus-related memory antibody repertoires could be used to define COVID-19 clinical manifestations predictive of clinical outcomes, we utilized a gradient boosting machine learning approach, XGBoost, to build a COVID-19-related virus exposure signature (COVID-VES) corresponding to the severity of patients' conditions in the discovery cohort, which follows a strategy used previously 21.

**Figure 4:**
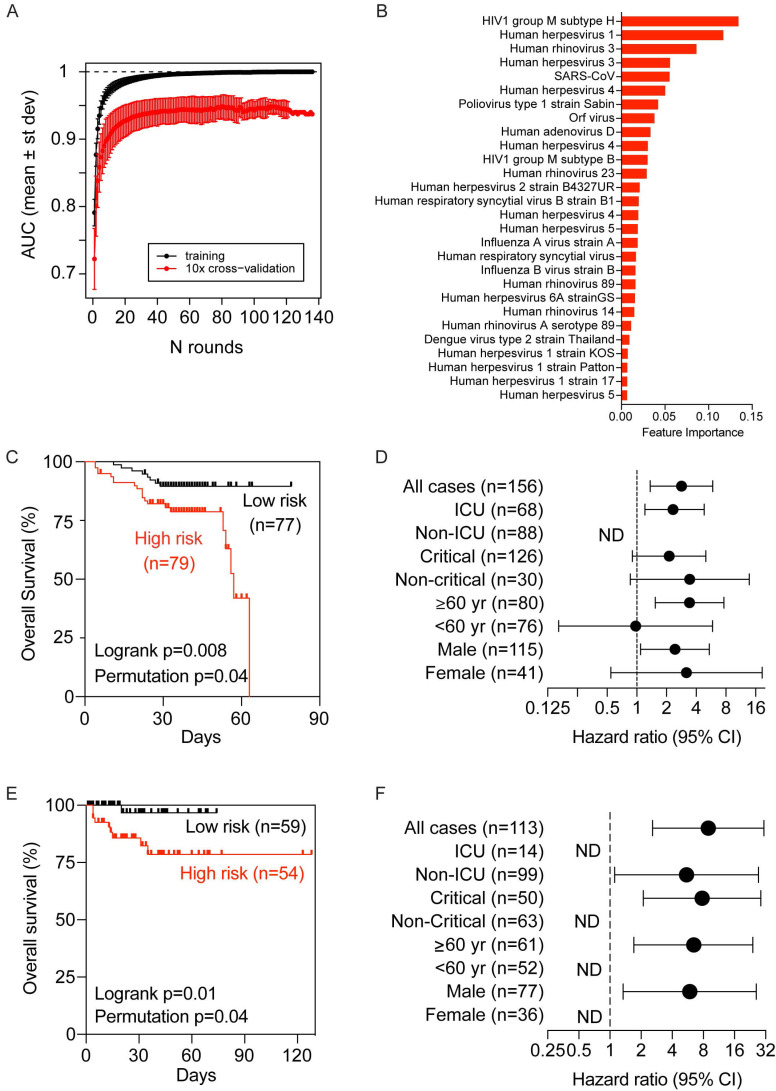
**Development and validation of a viral exposure signature predictive of disease severity.** (**A**) XGBoost with 10-fold cross validation for 100 iterations of balanced input data generated by ROSE. Each iteration showing the AUC value of training and cross validation sets. (**B**) The COVID-VES signature consisted of 28 viral strains that were selected in at least 50 of the 100 iterations predicted by XGBoost. (**C** and **E**) Survival risk predictions based on the COVID-VES signature in low- and high-risk patient groups in the discovery (**C**) and validation (**E**) cohorts, respectively. Survival time was based on days since admission. (**D** and **F**) Results from Cox proportional hazards regression analyses in the discovery (**D**) and validation (**F**) cohorts, respectively. Patients within each clinical group were classified into low- and high-risk categories based on the COVID-VES, then Cox proportional hazards ratios were determined. ND, not determined. P-values were determined with Logrank and Student's t-test. Error bars represent 95% confidential intervals.

We only included baseline samples (i.e., the first sample available upon hospitalization) to search for COVID-VES in order to minimize the potential disturbance of serological responses due to either the acute infection by SARS-CoV-2 or the length of hospitalization. Patients with a moderate to severe condition were compared to those with a critical condition. A total of 100 iterations were performed by applying the algorithm ROSE to generate balanced classes; for each iteration, XGBoost with 10-fold cross validation was found capable to significantly discriminate moderate samples from critical samples with area-under-the-curve (AUC) performance values close to 1 during training and above 0.9 during cross-validation (Figure 4A). The resulting COVID-VES signature consisted of 28 viral strains that were selected in at least 50 of the 100 iterations. All of these viral strains were positively associated with patients with critical condition (Figure 4B). We then tested if the resulting COVID-VES was associated with overall survival by applying the survival risk prediction algorithm successfully used in previous studies 26, 27. The survival risk prediction based on 10-fold cross-validation predicted patients into low- and high-risk groups with a significant difference in survival, as shown in the Kaplan-Meier plot (Figure 4C), yielding the log-rank P-value = 0.008. The resulting cross-validated misclassification rates were significantly lower than expected by chance (permutation P-value=0.04 based on 100 random permutations). The Cox proportional hazards regression analysis was also stratified by several clinical subgroups. COVID-VES risk was significantly associated with overall survival within the ICU, older (age ≥ 60 years), and male subcohorts (Figure 4D). The small size of our cohort, however, did not allow us to perform multivariable Cox regression analysis. To further validate the clinical utility of our COVID-VES, we performed survival risk prediction of this signature in 113 patients with survival data from the validation cohort. We found that COVID-VES was able to discriminate high-risk and low-risk patients with a significant difference in overall survival (Figure 4, E and F).

As the demographic factors such as age and sex could contribute to disease severity, we also examined the age and sex distribution in the two risk groups described in Figure 4C and E and found there were no statistically significant differences between the two risk groups (data not shown). These results suggest that predicted risk differences are mainly driven by the history of viral exposure rather than by differences in demographic factors.

**Figure 5:**
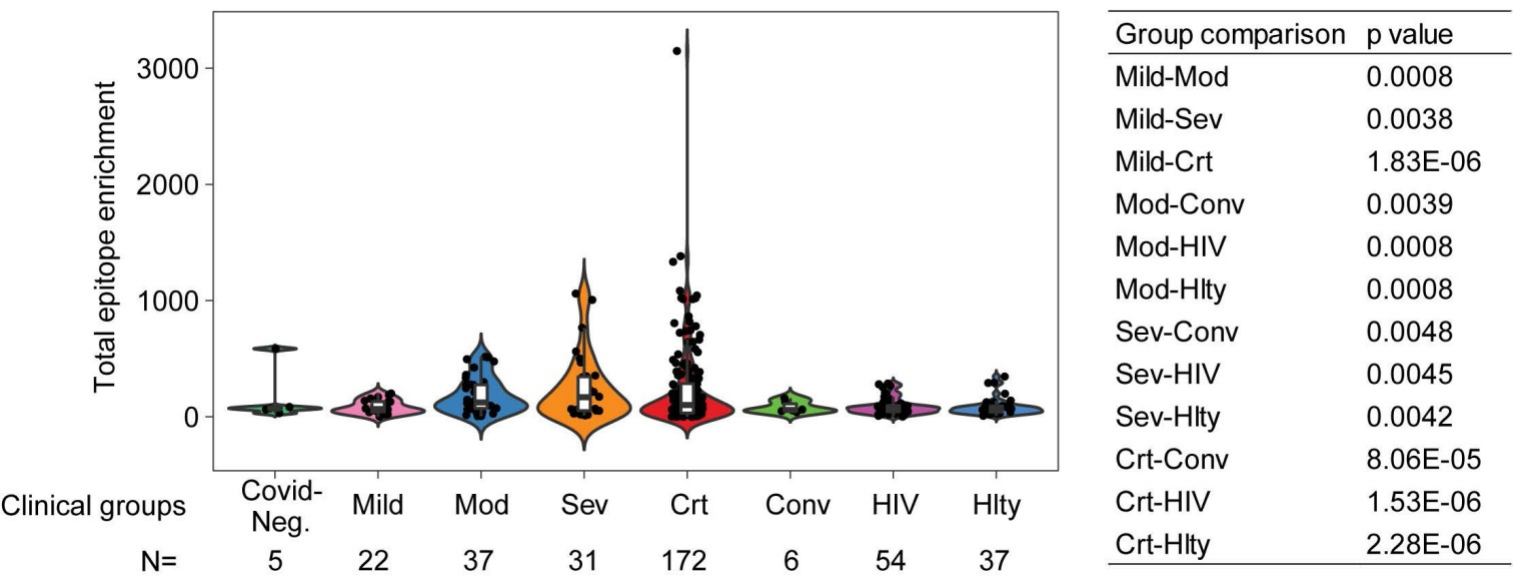
** Total epitope enrichment comparison in COVID-19 and HIV-1-infected patients.** Log-transformed total enrichment across all epitopes in the combined discovery and validation cohorts of COVID-19 patients from Northern Italy, and a cohort of HIV-1-infected (n=54) and healthy subjects (n=37) from NIH, USA. Significant p-values (<0.05) from the pairwise comparison of all patient groups are shown in the right-hand side panel. Covid-Neg, Covid negative; Mod, Moderate; Sev, Severe; Crt, Critical; Conv, Convalescent; Hlty, Healthy subjects. Box plots represent 25^th^ to 75^th^ percentiles and whiskers extend to 10^th^ and 90^th^ percentiles. P-values were determined with Student's t-test.

As noticed above, one of the limitations of the sequentially enrolled discovery cohort is that the patient numbers in different clinical severity groups are imbalanced, with only 12 moderate and 19 severe but 128 critical patients, and only 3 COVID-19 negative patients. To ameliorate this limitation, we combined the discovery and validation cohorts, thus improving the balance across mild (n=22), moderate (n=37), severe (n=31), and critical (n=172) COVID-19 positive patients. We also included 5 COVID-19 negative and 6 convalescent patients. There was a statistically significant increase in total reactive antibodies in COVID-19 positive patients correlated with clinical severity and, moreover, statistically significant increases in various strata of COVID-19 positive patients (namely, moderate, severe, and critical) compared to convalescent patients for COVID-19 (Figure 5). To further validate that the elevated immune memory antibody repertoires were modulated upon acute infection by SARS-CoV-2, we compared COVID-19 patients to another cohort of patients including 54 HIV-1-infected subjects on antiretroviral therapy and 37 healthy control patients enrolled at the NIH Clinical Center (Supplemental Table 2) and profiled with VirScan concurrently with COVID-19 patients. As shown in Figure 5, the HIV-1-infected group did not show increased antibody levels compared to its demographically matched healthy control group. Both groups appear to have total epitope enrichment levels significantly lower than those of COVID-19 positive subjects (Supplemental Table 3).

To further examine a potential bias of COVID-VES due to an initial search based on unbalanced numbers of patients with moderate and critical conditions in the discovery cohort, we performed XGBoost with ROSE on a combined discovery and validation cohort. Using the same feature selection criteria, we found 35 significant viral strains that could discriminate mild/moderate from critical cases (Supplemental Figure 5A-B). Most of them overlapped with COVID-VES. Despite some differences in the feature importance with the XGBoost modeling, this alternative COVID-VES showed good performance to capture the low- vs high-risk overall survival differences of the stratified cohort (log rank p<0.01; permutation p<0.01) (Supplemental Figure 5C).

## Discussion

Humoral immunity plays an important role in antiviral response by producing antibodies against various pathogens such as SARS-CoV-2, among others, which may result in convalescence.

Using VirScan, we have determined the exposure history of COVID-19 patients to most known human viruses. Our findings suggest that the dysregulation or imbalanced activation of humoral immunity may be associated with poor COVID-19 outcomes. A surprising finding of this study is a marked increase in the overall immune memory antibody repertoire activity in COVID-19 patients linked to the trajectories of disease severity. This conclusion is supported by the following observations. First, levels of total reactive antibodies against unique epitopes of known viruses were much higher in hospitalized COVID-19 patients than in non-COVID-19 patients who were also hospitalized due to pneumonia. Second, COVID-19 patients in critical condition had much higher levels of reactive antibodies to known viruses than those in severe or moderate condition. These results suggest that COVID-19 patients in critical condition may have a uniquely different host immune response and presumably a different host genetic background. This view is consistent with a recent genome-wide association study identifying a 3p21.31 gene cluster as a genetic susceptibility locus in patients with COVID-19 with respiratory failure 28 and with the recent identification of monogenic defects of type I interferon immunity in patients with critical COVID-19 29. Third, longitudinal analysis revealed that during their hospitalization, patients in critical condition showed the highest elevation of reactive antibodies to known viruses compared to patients in severe or moderate condition. It appears that the elevated immune memory antibody repertoire activity is associated with poor clinical trajectories in COVID-19 patients. Levels of antibodies against a single linear epitope corresponding to SARS-CoV spike protein with 100% homology to SARS-CoV-2 were also linked to trajectories of disease severity. There was no significant difference in immune memory antibody repertoires between older or younger patients but a small difference between men and women who had acute infections of SARS-CoV-2. This is in contrast to the observations that both male and older individuals are more likely to experience severe COVID-19-related symptoms than female or younger individuals 30. Our data suggest that SARS-CoV-2 may directly stimulate an individual's overall immune memory antibody repertoire activity. These results are unexpected since humoral immunity is thought to be very stable over time due to long-lived plasma cells since long-term antibody responses are critical for protective immunity against pathogens 31-33. Interestingly, a recent study revealed that measles virus infection can diminish preexisting antibodies that offer protection from other pathogens, which may in turn create potential vulnerability to future infections 23. It appears that COVID-19 acute infection and measles virus infection may follow different molecular mechanisms to alter humoral immune memory. Consistently, a recent study demonstrated that COVID-19 infection is associated with increased frequencies of proliferation of memory B cell subsets but no changes in naïve B-cell frequencies 34. The ability of SARS-CoV-2 to selectively promote proliferation of memory B cells could explain our findings of a marked increase in an overall immune memory antibody repertoire activity linked to poor clinical trajectories of COVID-19 patients. Future studies will explore whether dampening COVID-19-induced reactivation of memory B cells may be a viable strategy to control the clinical severity of COVID-19 patients. Encouraging results were obtained with the use of dexamethasone 35, a synthetic corticosteroid as a broad-spectrum immunosuppressor that can affect both T cells and B cells. Inhibition of Bruton tyrosine kinase, a regulator of B-cell maturation 36, could be another viable therapeutic strategy to improve clinical outcomes of COVID-19 patients 37.

Autoimmune diseases are complex disorders resulting from the failure of immunologic tolerance leading to an immune response against the host antigens. Autoimmune reactions signify an imbalance between effector and regulatory immune responses 38. They may arise from a combination of genetic and environmental factors. Viral infection is considered as an environmental factor to trigger autoimmune disease 39. Examples of human viruses that may precipitate autoimmune manifestations include HHV-4 (EBV) and HHV-6 linked to multiple sclerosis 40, 41, parvovirus B19 linked to rheumatoid arthritis 42, and hepatitis C virus linked to cryoglobulinemia 43. Several recent studies suggest a possible involvement of SARS-CoV-2 infection in inducing autoimmune and autoinflammatory manifestations such as multisystem inflammatory syndrome in children (MIS-C) 44, 45. It is plausible that various clinical manifestations of COVID-19 may be the result of SARS-CoV-2-induced autoimmunity. While how COVID-19 induces autoimmunity remains unclear, molecular mimicry has been suggested as a plausible mechanism 39. This mechanism suggests the presence of similar antigens between viruses and hosts to facilitate pathogens to avoid the host immune response and is mainly mediated via a T-cell response 39. However, our results suggest that COVID-19 may affect B-cell repertoires as we observed a marked elevation of memory antibodies to past history of viral infection of most known viruses, regardless of viral types. These results are consistent with the idea that SARS-CoV-2 may directly activate memory B cells, a concept supported by a recent observation that COVID-19 infection is mainly associated with increased frequencies of proliferation of memory B-cell subsets and expansion of plasmablasts 34. It is possible that memory B cells are much more sensitive than naïve B cells to SARS-CoV-2 and that patients with activation of certain memory B cell subsets may be more vulnerable to COVID-19-induced disease severity. This could explain our finding that HCV infection was seen exclusively in hospitalized Brescia COVID-19 patients with a critical condition. Although speculative, it will be interesting to determine if HCV patients who have achieved a sustained virologic response by anti-HCV therapy are still vulnerable for COVID-19-mediated disease severity. Additional prospective studies with larger cohorts are warranted to test these hypotheses.

VirScan is a powerful technique to profile the humoral response induced by infectious diseases. During the preparation and peer-review of this manuscript, other studies using the VirScan technology have been published showing consistent findings. In one of these studies, Shrock et al 46 updated the VirScan virome phage library including SAS-CoV-2 epitopes which was used for serological profiling of COVID-19 patient and pre-COVID-19 era control sera. It showed that COVID-19 patients mounted a strong antibody response to specific regions of the spike and nucleocapsid proteins of SARS-CoV-2, similar to the findings reported here. Interestingly, this study revealed that serum samples from COVID-19 patients also exhibited a significantly higher level of reactivity to seasonal HCoV peptides compared to pre-COVID-19 era controls, which is speculated to be likely due to an anamnestic response boosting B cell memory against HCoVs. In another study, Zamecnik et al 47 introduced ReScan, a modified version of VirScan, which combines paper-based microarrays and programmable phage display and was used to screen for and isolate the most immunogenic peptides for SARS-CoV-2 antibody diagnostics. In excellent agreement to our findings, this study isolated epitopes within the spike and nucleocapsid proteins with a strong immunogenic reactivity, which are the same as those reported here.

In summary, by determining serological responses to history of infection from most known human viruses, we linked the activity of memory antibody immune repertoires to clinical manifestations of COVID-19 patients. We developed a COVID-19-related viral exposure signature as serological biomarkers that may be useful to identify COVID-19 patients who may progress to autoimmune and autoinflammatory disease that may require tailored treatments. The main limitations of this study are the inclusion of only 284 COVID-19 patients with imbalanced number of patients in the discovery cohort and only a few SARS-CoV-2 negative acute infectious pneumonia control patients. While we observed a consistent and clear elevation of individual immune memory antibody repertoires linked to trajectories of disease severity over time, there remains a substantial heterogeneity within each patient group, which will require expanding our analysis in the future to larger cohorts with well-balanced severity groups controlled by SARS-CoV-2 negative acute infectious pneumonia control patients and increased time resolution in order to gain a deeper understanding of the complex interplay between host immunity and SARS-CoV-2 infection.

## Methods

*Study cohorts.* Sera were prepared from blood donated by patients admitted in hospitals in Italy due to suspected COVID-19 since March 2020. We first analyzed sequentially collected blood samples of 159 patients from Brescia, Italy in March 2020 when Northern Italy was hit by the first wave of COVID-19, as a discovery cohort. We then analyzed additionally collected blood samples from 125 patients from Brescia, Monza and Pavia, Italy from April to May 2020, when the stress on the healthcare system was relieved, as an independent validation cohort. Patients were classified into categories of asymptomatic, mild, moderate, severe, and critical according to clinical features (National Health Commission & National Administration of Traditional Chinese Medicine, 2020). Specific definitions of clinical severity are: (1) Asymptomatic (individual who tested positive by qRT-PCR but did not develop symptoms); (2) Mild (modest symptoms and no pneumonia); (3) Moderate (fever and respiratory symptoms plus radiological evidence of pneumonia; use of low-flow oxygen is still part of this category but O2 saturation is >93% at rest); (4) Severe (oxygen saturation at rest of 93% or lower OR respirate rate >30/min OR PaO2 FiO2 <300 mmHg; use of oxygen is still part of this category); (5) Critical (any of the following: mechanical ventilation via CPAP, BiPAP, intubation, or high-flow oxygen; septic shock; organ damage requiring admission in the ICU); (6) Convalescent (tested positive at the time of hospital admission, then negative at the time of blood sample collection). Patient clinical information and eligibility were surveyed with the standard COVID-19 Human Genetic Effort Patient Screen form. Sera were isolated from whole blood for analysis. Sera from HIV-1-infected individuals on antiretroviral therapy and from anonymous healthy blood donors were collected after informed consent was obtained under the NIH IRB-approved protocols NCT02081638 and NCT00001846, respectively.

*VirScan T7 phage epitope library.* The sera samples from enrolled patients were used to perform high through-put phage-immunoprecipitation (Phage-IP; VirScan) with a T7 phage library expressing epitopes of humoral virome. We used the v2.0 epitope library which consists of 96,179 viral peptides generated from the human virome of all 1,276 known humoral virus strains 22. In this phage library, there are 3,663 epitopes from 16 species of coronaviruses, including 80 epitopes from SARS-CoV. The virome was tiled to 56 aminoacid long epitopes with 28 aminoacid overlaps. The epitope tiling was then cloned to a T7 phage library for displaying. Phage library was amplified using BLT5403 E. coli strain with the plate lysate method previously described 22. The phage library quality was assessed in two ways. First, it was titrated with standard protocol to ensure the titer of phage is about 1x10^11^pfu/mL. Second, a 10μl aliquot of the amplified phage library was lysed by boiling at 95°C for 10 min followed by two steps of PCR to amplify and index the cloned epitope sequences in the phage genome. The constructed sequencing library was then sequenced at the NCI CCR Frederick Sequencing Facility. The phage library passed quality control as a total coverage of more than 99.99% of the designed epitopes was achieved. These sequencing results also serve as input for downstream analysis of phage-IP-seq.

*Phage-IP-seq***.** Phage-IP was then performed using collected sera samples and phage library as previously described 21. The day before phage-IP, 96-deep-well plates (BradTech, Catalog #EW-07904-04) were blocked using bovine serum albumin in TBST buffer. After overnight incubation, the blocking buffer was aspirated and sera samples containing 2 μg of total IgG were mixed with 2x10^10^ pfu T7 library in 1mL dilution buffer (20 mM Tris-HCl, pH 8.0, 100 mM NaCl, 6 mM MgSO4) supplemented with 50 μg/mL chloramphenicol and 50μg/mL kanamycin into the deep well plate, and rotated at 4°C for 20 hours for the phage to form complex with the antibodies from sera. Technical duplicate plates were introduced during this step. After overnight complex formation, 20 μl protein A and 20 μl protein G Dynabeads (Thermo Fisher, Catalog #10008D and #10009D) were added to each well containing the phage and antibody complex, and rotated for another 4 hours at 4°C. The finally formed Dynabeads-antibody-phage complex was then washed three times with washing buffer (50 mM Tris-HCl, pH7.5, 150 mM NaCl, 0.1% NP-40) to eliminate the non-specific binding. After wash, the Dynabeads with antibody-phage complex were suspended in 40 μl ultrapure water and transferred into a 96-well PCR plate, followed by boiling at 95°C for 10min to recover the phage genomic DNA. The recovered phage genomic DNA was used as template of first round PCR to amplify the epitope expressing sequences. A second round PCR was employed to add index barcode to the DNA product of first round PCR of each sample well on the 96-well plate. The products of second PCR were then pooled together and loaded to a 2% agarose gel and target size fragment were cut and recovered using a gel extraction kit (Qiagen, Catalog #28704). With this, the DNA sequencing library was successfully constructed. Recovered sequencing library now contained barcoded DNA fragments encoding viral epitopes that were recognized by antibodies in the sera. This DNA library was then sequenced the same way as phage epitope library did, on an Illumina NextSeq 500 platform with 75bp single read, at NCI CCR Frederick Sequencing Core Facility. A total of 200 million reads per lane were obtained with an average of one million reads per serum sample and minimum 90% mapping rates were achieved.

*Sequencing data processing and informatic epitope score analysis.* Raw data were demultiplexed with BCL2FASTQ2 and converted to fastq format. The fastq files were mapped to the virome library sequence with Bowtie. To call hits, we first calculated P-value of each epitope by fitting the observed post-alignment read count abundance distribution of phage-IP enriched epitopes in to a zero-inflated Generalized Poisson null distribution regression model. Then initial QC was performed by using scatterplots of the -log10(P-values) and a sliding window width of 0.005 from 0 to 2 across the axis of one replicate. A threshold of -log10(P-value) was set between 2.3 and 4.7 in both replicates based on -log10(P-value) distribution to correct batch effect and background noises. A hit was called to reference epitope if present in both technical replicate samples. Hits that were present in less than two sera samples or more than three mock immunoprecipitation wells were eliminated as background noise. The called epitope hits were then grouped to the virus it is derived. Another threshold was also used to bioinformatically remove cross-reactive antibodies: called viruses were sorted by total hits number in descending order, and then iterate through each virus in this order to remove any called epitope that shares more than seven amino acids homology with epitopes of previous virus in this list. All left epitopes and virus are now used as specific signal of each sample. The number of epitope hits of each virus after phage-IP are summed and used as the so-called feature score of each virus. As a complementary epitope binding quantitation strategy and to overcome possible limitations of the feature score calculation, we also employed a recently developed strategy to calculate z-scores, by comparing antibody-enriched libraries to replicate negative controls (mock IPs). By determining the observed abundance excess relative to the background, divided by the background signal's standard deviation, and then converting to log scale, we assessed the EBS.

*Epitope abundance count and virus prevalence.* Epitopes with a feature score greater than one were counted as unique epitopes. Any virus with more than one non-cross-reactive epitope detected is counted as positive. Detected viruses are then counted at strain level according to epitopes detected and grouped to species level. Unique epitopes and grouped detected virus species are then counted in each sample to plot the unique epitope abundance and virus prevalence. Number of detected SARS-CoV epitope are extracted and used for heatmap plot and subsequent analysis.

*B-cell epitope antigenicity prediction.* Sequences of SARS-CoV spike and nucleocapsid proteins were used for prediction of B-cell antigenicity with the B-cell epitope prediction tool of the Immune Epitope Database (http://tools.iedb.org). A B-cell antigenicity score of 1-100 for each epitope was predicted by the online software.

*LIPS assays for measurement of SARS-CoV-2 spike and nucleocapsid antibodies.* Liquid-phase immunoassay technology, a luciferase immunoprecipitation system (LIPS) assay was used to detect SARS-CoV-2 spike and nucleocapsid antibodies as previously described 24. Briefly, viral proteins fused to light-emitting luciferase are immunoprecipitated with patient serum. The SARS-CoV-2 nucleocapsid was constructed in the standard pREN2 vector as a C-terminal Renilla luciferase fusion protein and the spike protein was generated as an N-terminal fusion with the Gaussia luciferase in the pGAUS3 vector. The nucleocapsid and spike protein-light emitting plasmid constructs were then transfected into Cos1 cells and crude cell lysate were prepared 48 hour later. For antibody measurement, heat-inactivated serum/plasma samples were diluted 1:10 in assay buffer A (20 mM Tris-Cl, pH 7.5, 150 mM NaCl, 5 mM MgCl_2_, and 1% Triton X-100), and 10 µL of the diluted sample was then mixed with 1x10^7^ light units (LU) Cos1 crude cell lysate in 50 μL of buffer A. After incubation for 1 hour, the mixture was transferred to a microtiter filter plate containing protein A/G beads and another 1-hour incubation. Microtiter plates containing the beads were then washed 8 times with buffer A and twice with phosphate-buffered saline to remove unbound antigens. After the final wash, coelenterazine substrate (Promega) was added to detect the luciferase activity, and light units (LU) were measured using a Berthold LB 960 Centro microplate luminometer (Berthold Technologies).

*Sequence alignment.* Protein sequences of SARS-CoV and SARS-CoV-2 are aligned with the EBI Clustal Omega Multiple Sequence Alignment tool (https://www.ebi.ac.uk/Tools/msa/clustalo/) and NCBI BLASTP tool (https://blast.ncbi.nlm.nih.gov/). Cosmetic labeling was made to highlight the region of interest in the sequences.

*Analysis of longitudinal EBS progression.* The normalized EBS signal across all epitopes was analyzed longitudinally for all individuals with two or more timepoints. For those with two timepoints, the normalized EBS signal was linearly interpolated. For those with more than two timepoints, data were fitted using LOESS (Locally Estimated Scatterplot Smoothing) regression. The fitted curves were then averaged within patient groups. Linear regression was performed on the averaged profiles to extract the slope's value and standard error.

*XGBoost regression of viral exposure signature.* To extract the viral exposure signature associated with the clinical severity of COVID-19 patients, we employed the open source software XGBoost, which implements machine-learning algorithms under a parallel gradient tree boosting framework. Since the clinical severity classes were strongly imbalanced, we preprocessed the datasets using the ROSE (v. 0.0-3) R package, which stochastically generates balanced samples according to a smoothed bootstrap approach. By implementing ROSE, we generated 100 iterations of balanced datasets consisting of moderate (n=70) and critical (n=70) groups, which were subsequently used for XGBoost regression. We used grid search strategy in XGBoost to maximize the computed mean AUC value. The AUC was generated with 10-fold cross validation where 90% of samples were used for training and the remaining 10% of samples were used as independent validation. To avoid overfitting, we set the early stop of model training to at least 20 rounds when no incremental improvement was observed in the AUC. The data attributes such as virus epitope expression and patient meta data were assigned scores that indicate each attribute's importance in the construction of the boosted decision trees, allowing features to be ranked and compared to each other, and subsequently, feature importance scores were averaged across all decision trees within the model. The final output of XGBoost resulted then in an Importance Score (Feature Importance) to each feature (virus). This Importance Score quantifies the improvement in accuracy brought by a feature to the decision tree branches it is on during the tree boosting and grid search process. The higher score, the more importance of the feature (virus) to the module prediction. With this optimized iteration model, XGBoost conducted feature selection and output viral exposure signatures consisting of 56 viruses, which could discriminate the critical versus the moderate group of COVID-19 patients across all 100 iterations. Among the 56 viruses, 28 were the common viruses that were predicted at least 50% of the 100 iterations.

*VES-based survival prediction.* We used the 28 viral strain exposure signature (referred to as COVID-VES) to predict survival in the Brescia cohort. The survival risk prediction tool of the BRB-ArrayTools Stable Version 4.6.1 was used for survival analysis. Two risk groups at median cut off were selected with 10-fold cross validation method and 100 permutations were performed. With a permutation P-value 0.03, the output survival risk prediction of the 156 training samples were then used for survival analysis for different subgroups of the cohorts with GraphPad Prism 8. Hazard ratio and 95% confidential interval was used for forest plot of the different groups.

*Statistics.* Statistical differences and significances between patient groups of moderate, severe and critical are examined with XGBoost algorithm and survival analyses were done with GraphPad Prism 8. P-values were calculated with Student's t-test or log-rank t-test. All plot figures were generated with GraphPad Prism 8, R3.5.3 or R3.5.1 based BRB-ArrayTools Stable Version 4.6.1. All other analyses were performed with GraphPad Prism 8 and R 3.6.3. Comparison of different groups were analyzed with Fisher's exact tests and two-sided Student's t-tests.

*Study Approval.* De-identified blood samples were obtained under protocol NP-4000 approved by Comitato Etico Provinciale, Brescia, Italy; protocol 84/2020 COVID-STORM approved by the Ethics Committee of the Italian National Institute of Infectious Diseases “Lazzaro Spallanzani”; and protocol 20200037677 approved by the IRB of Fondazione IRCCCS Policlinico San Matteo, Pavia, Italy. Protocols NCT02081638 and NCT00001846 approved by the IRB committee of the National Institutes of Health, Bethesda, Maryland.

## Supplementary Material

Supplementary figures and tables.

## Figures and Tables

**Table 1:** Clinical characteristics of hospitalized patients with pneumonia from Italy

Clinical Variable	Discovery (Brescia) (n=159)	Validation (Brescia/Monza/Pavia) (n=125)	*p* value^f^
** SARS-CoV-2 status^a^ - no. (%)**			0.86^g^
Negative	3 (2)	2 (2)	
Positive	156 (98)	114 (91)	
Convalescent	0	6 (5)	
No data	0	3 (2)	
** Sex - no. (%)**			0.30
Male	116 (73)	84 (67)	
Female	43 (27)	41 (33)	
Age - median (range)	60 (23-89)	63 (0.1-95)	0.25^g^
** Location - no.**			NA^h^
Brescia	159	60	
Monza	0	31	
Pavia	0	34	
** Sample time point^b^ - no.**			0.74^g^
T0	159	125	
T1	76	62	
T2	41	27	
T3	22	12	
T4	0	5	
T5	0	3	
** Clinical Severity^c^ - no.**			<0.001
Moderate	12	56^e^	
Severe	19	13	
Critical	128	56	
** HIV status - no. (%)**			0.31
Postitive	7 (5)	2 (2)	
Negative	152 (95)	123 (98)	
** Immunodeficiency^d^ - no. (%)**			0.60
Yes	23 (14)	15 (12)	
No	136 (86)	110 (88)	
** Dexamethasone treatment - no. (%)**			<0.001
Yes	59 (37)	10 (8)	
No	100 (63)	115 (92)	
** Outcome - no. (%)**			0.01
Alive	129 (81)	111 (89)	
Deceased	30 (19)	11 (9)	
No data	0	3 (2)	
** Enrollment date**	March 2020	Apr-May 2020	

^a^SARS-CoV-2 status was determined by quantitative real-time reverse-transcriptase-polymerase-chain-reaction (qRT-PCR) assay of nasal and pharyngeal swab specimens and/or by positive serology tests. ^b^Longitudinal time points of blood collection are indicated as T0 - T5. T0, the first blood collection; T5, the last blood collection. ^c^Definition details in Materials and Methods. ^d^Including primary and secondary immunodeficiency. ^e^Moderate (n=56) combined 1 asymptomatic, 6 Convalescent, 21 mild and 28 moderate cases. ^f^Fisher's exact test, unless specified. ^g^Unpaired t-test. ^h^Not available
