# Supplementary material for: Serological responses to human virome define clinical outcomes of Italian patients infected with SARS-CoV-2
Source: Int J Biol Sci. 2022 Sep 1;18(15):5591–606. doi: 10.7150/ijbs.78002 (PMC9576512; doi:10.7150/ijbs.78002)
Supplement: Supplementary file 1 — Supplementary figures and tables. [file ijbsv18p5591s1.pdf]

Supplemental Table 1. Viral prevalence in Brescia cohort. Related to Figure 1.

Virus species	Total (n=156)	Moderate (n=11)	Severe (n=19)	Critical (n=126)
Human respiratory syncytial virus	81%	64%	79%	83%
Human herpesvirus 4	74%	91%	95%	70%
Human herpesvirus 1	67%	55%	79%	66%
Human herpesvirus 5	55%	55%	63%	54%
SARS-CoV	48%	27%	53%	49%
Rhinovirus B	37%	27%	58%	34%
Rhinovirus A	33%	27%	47%	32%
Human immunodeficiency virus 1	23%	27%	32%	21%
Human herpesvirus 3	22%	9%	32%	22%
Influenza B virus	19%	9%	16%	21%
Human adenovirus C	17%	9%	21%	17%
Enterovirus B	14%	0%	16%	15%
Enterovirus C	13%	0%	16%	14%
Dengue virus	13%	9%	21%	12%
Human herpesvirus 6B	13%	9%	11%	13%
Mamastrovirus 1	10%	9%	11%	10%
Orf virus	10%	0%	16%	10%
Betacoronavirus 1	10%	0%	11%	10%
Rotavirus A	10%	0%	11%	10%
Enterovirus A	8%	0%	16%	7%
Influenza A virus	8%	9%	0%	9%
Human adenovirus D	7%	18%	0%	7%
Human coronavirus NL63	7%	0%	11%	7%
Human herpesvirus 8	6%	9%	0%	7%
Norwalk virus	6%	0%	21%	5%
Hepatitis B virus	6%	0%	11%	6%
Hepatitis C virus	6%	0%	0%	7%
Human herpesvirus 2	6%	9%	0%	6%
Human coronavirus 229E	4%	0%	5%	4%
Alphapapillomavirus 9	3%	0%	5%	3%

Supplemental Table 2. Clinical characteristics of NIH HIV patients. Related to Figure 5.

Clinical Variable	ALL (n=91)	HIV (n=54)	Healthy (n=37)
**Age - year**			
Median (range)	54 (22-87)	55 (32-65)	51 (22-87)
Missing data	3	3	0
**Sex - no. (%)**			
Male	64 (70)	35 (65)	29 (78)
Female	24 (26)	16 (30)	8 (22)
Missing data	3 (3)	3 (6)	0

Supplemental Table 3. p-values among pairwise comparison groups. Related to Figure 5

comparison	mean of group1	mean of group2	p value
COVID negative-Mild	167.56	84.88	0.4763
COVID negative-Moderate	167.56	185.36	0.8761
COVID negative-Severe	167.56	246.43	0.5231
COVID negative-Critical	167.56	233.86	0.5700
COVID negative-Convalescent	167.56	82.50	0.4674
COVID negative-HIV	167.56	89.29	0.4980
COVID negative-Healthy	167.56	86.42	0.4841
Mild-Moderate	84.88	185.36	0.0008
Mild-Severe	84.88	246.43	0.0038
Mild-Critical	84.88	233.86	1.83E-06
Mild-Convalescent	84.88	82.50	0.9224
Mild-HIV	84.88	89.29	0.7831
Mild-Healthy	84.88	86.42	0.9316
Moderate-Severe	185.36	246.43	0.2841
Moderate-Critical	185.36	233.86	0.1945
Moderate-Convalescent	185.36	82.50	0.0039
Moderate-HIV	185.36	89.29	0.0008
Moderate-Healthy	185.36	86.42	0.0009
Severe-Critical	246.43	233.86	0.8278
Severe-Convalescent	246.43	82.50	0.0048
Severe-HIV	246.43	89.29	0.0045
Severe-Healthy	246.43	86.42	0.0041
Critical-Convalescent	233.86	82.50	8.06E-05
Critical-HIV	233.86	89.29	1.53E-06
Critical-Healthy	233.86	86.42	2.28E-06
Convalescent-HIV	82.50	89.29	0.7708
Convalescent-Healthy	82.50	86.42	0.8731
HIV-Healthy	89.29	86.42	0.8591

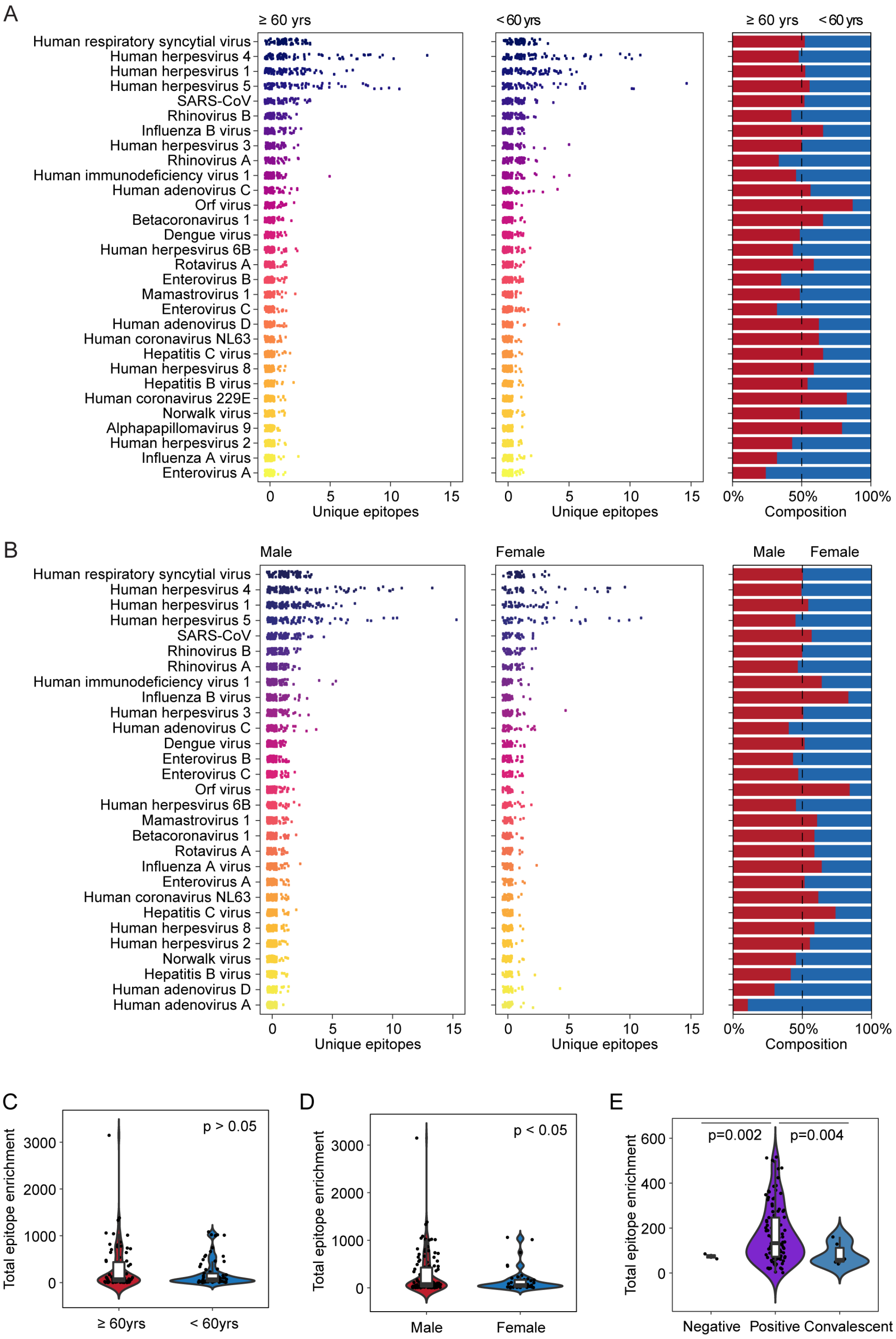

Supplemental Figure 1. **Prevalence of viral antibodies.** (**A**) Number of unique epitopes and composition of prevalence in cases with age above (≥ 60 yrs) and below (< 60 yrs) the median of

the discovery cohort. (**B**) Number of unique epitopes and composition of prevalence in male and female cases. (**C**) Total reactivity across all epitopes in cases with age ≥ 60 yrs vs age < 60 yrs. (**D**) Total reactivity across all epitopes in male and female cases. For each violin plot, the embedded box spans the interquartile range around the median (thick horizontal line), whereas the contour denotes the kernel density estimate of the distribution. (**E**) Total reactivity of all epitopes in COVID-19 negative, positive and convalescent groups in the validation cohort. Box plots represent 25th to 75th percentiles and whiskers extend to 10th and 90th percentiles. P-values were determined with Student's t-test.

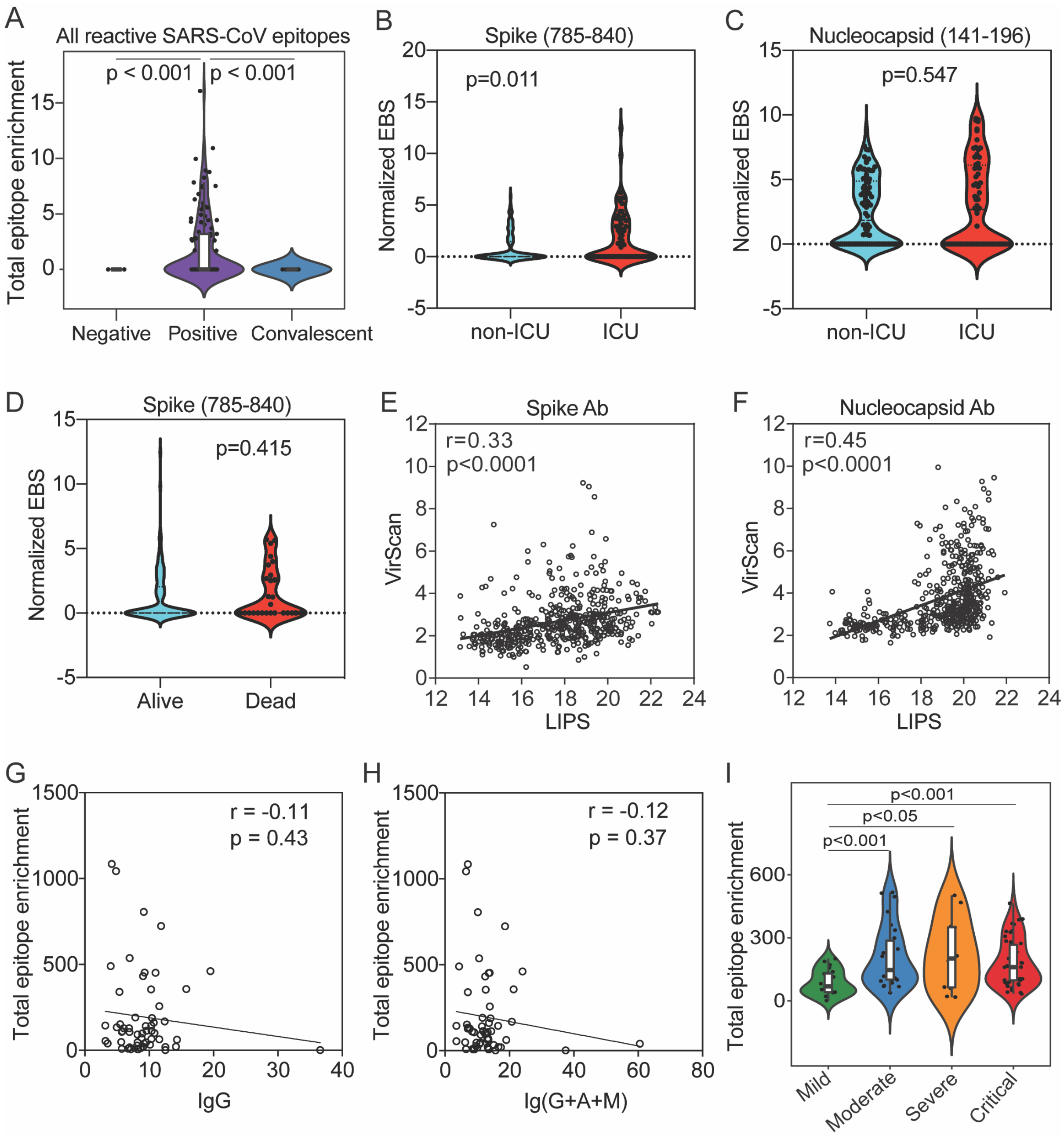

Supplemental Figure 2. Serological responses to SARS-CoV virus. (**A**) The response in COVID-19 negative, positive and convalescent patients in the validation cohort. (**B-D**) Serological responses in hospitalized patients with ICU (**B-C**) or death status (**D**). (**E-F**) Correlation of VirScan signal to the luciferase immunoprecipitation system (LIPS) assay results. (**G-H**) Correlation of the total reactivity of all epitopes to the immunoglobulin concentration measured by the ELISA assay. (**I**) The total enrichment of all epitopes in the mild, moderate, severe, and critical groups in the validation cohort. Log transformation was applied. In violin plots, the width of violin plots indicates the kernel density estimate of values; boxes span the interquartile range; lines within boxes represent the median. Box plots represent 25th to 75th

percentiles and whiskers extend to 10th and 90th percentiles. P-values were determined with Student's t-test.

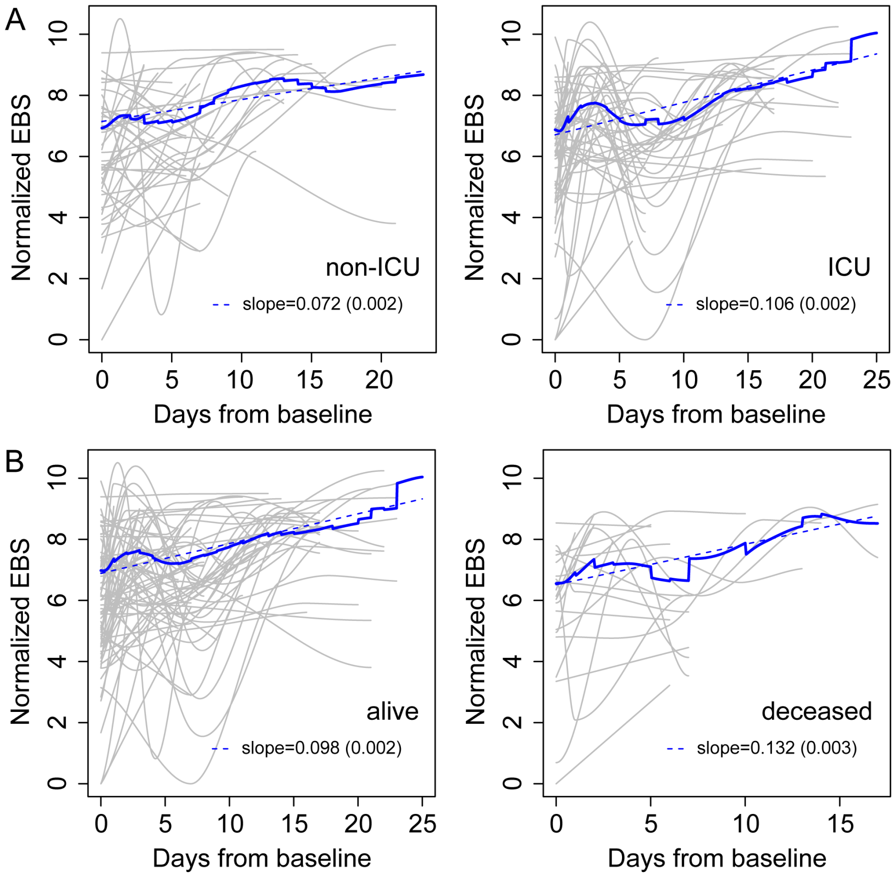

Supplemental Figure 3. Longitudinal progression of the normalized EBS across individuals. (**A**) Individual trajectories over time for patients grouped by hospital ward (gray lines), which were averaged (solid blue line) and fitted by linear regression (dashed blue line; slope and standard error shown in the legend). Baseline refers to the first sample obtained after admission to the hospital. Left: non-ICU; right: ICU. (**B**) Analogous results for patients grouped by clinical outcome. Left: alive; right: deceased. P-values were determined with Student's t-test.

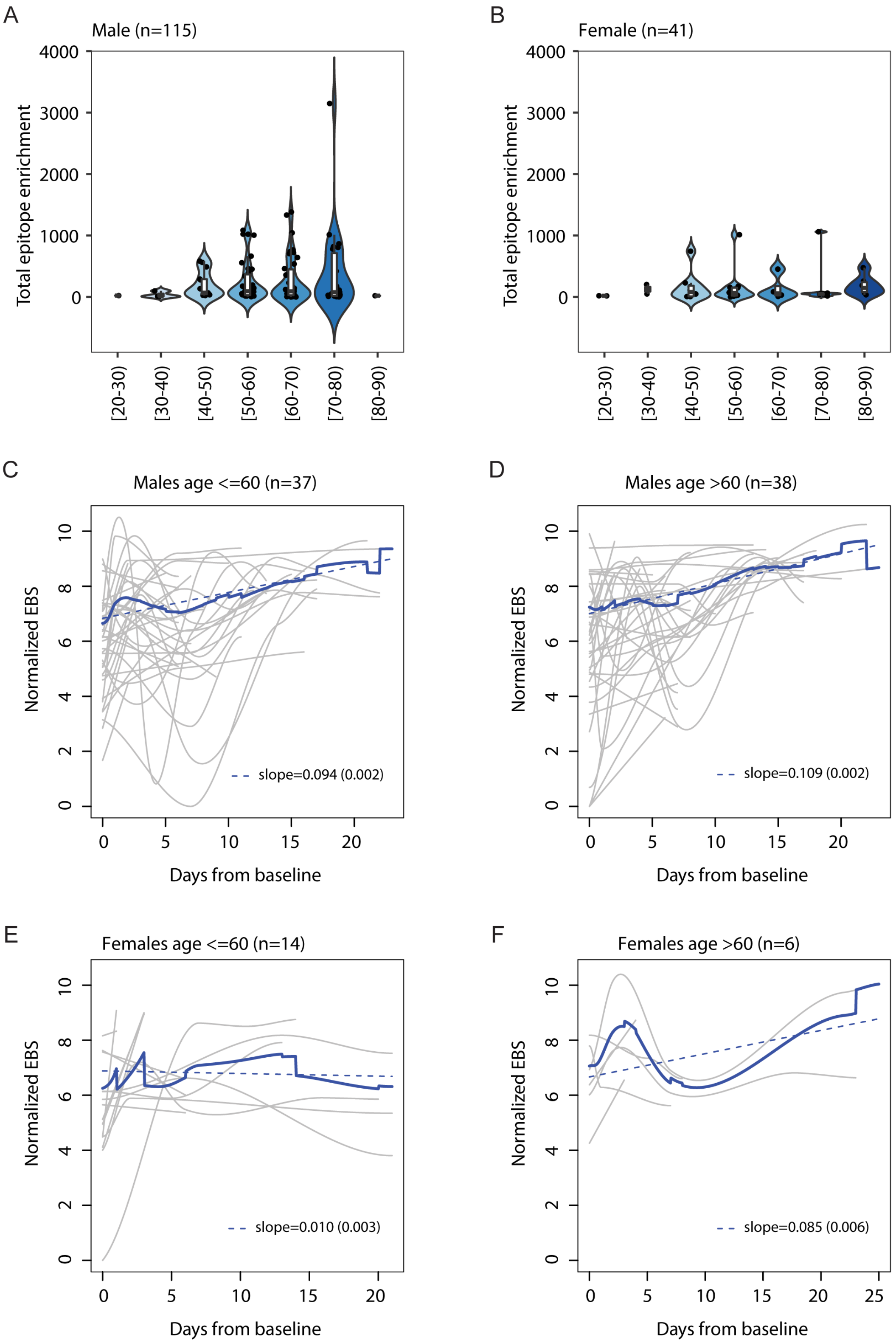

Supplemental Figure 4. **Sex and age effects of the humoral immune response of COVID-19 patients.** (**A-B**) Total epitope enrichment at baseline as a function of age for male (**A**) and

female (**B**) patients, respectively. (**C-F**) Longitudinal progression of the normalized EBS for younger males (**C**), older males (**D**), younger females (**E**), and older females (**F**), respectively. In violin plots, boxes span the interquartile range; lines within boxes represent the median; the width of violin plots indicates the kernel density of values. Box plots represent 25th to 75th percentiles and whiskers extend to 10th and 90th percentiles.

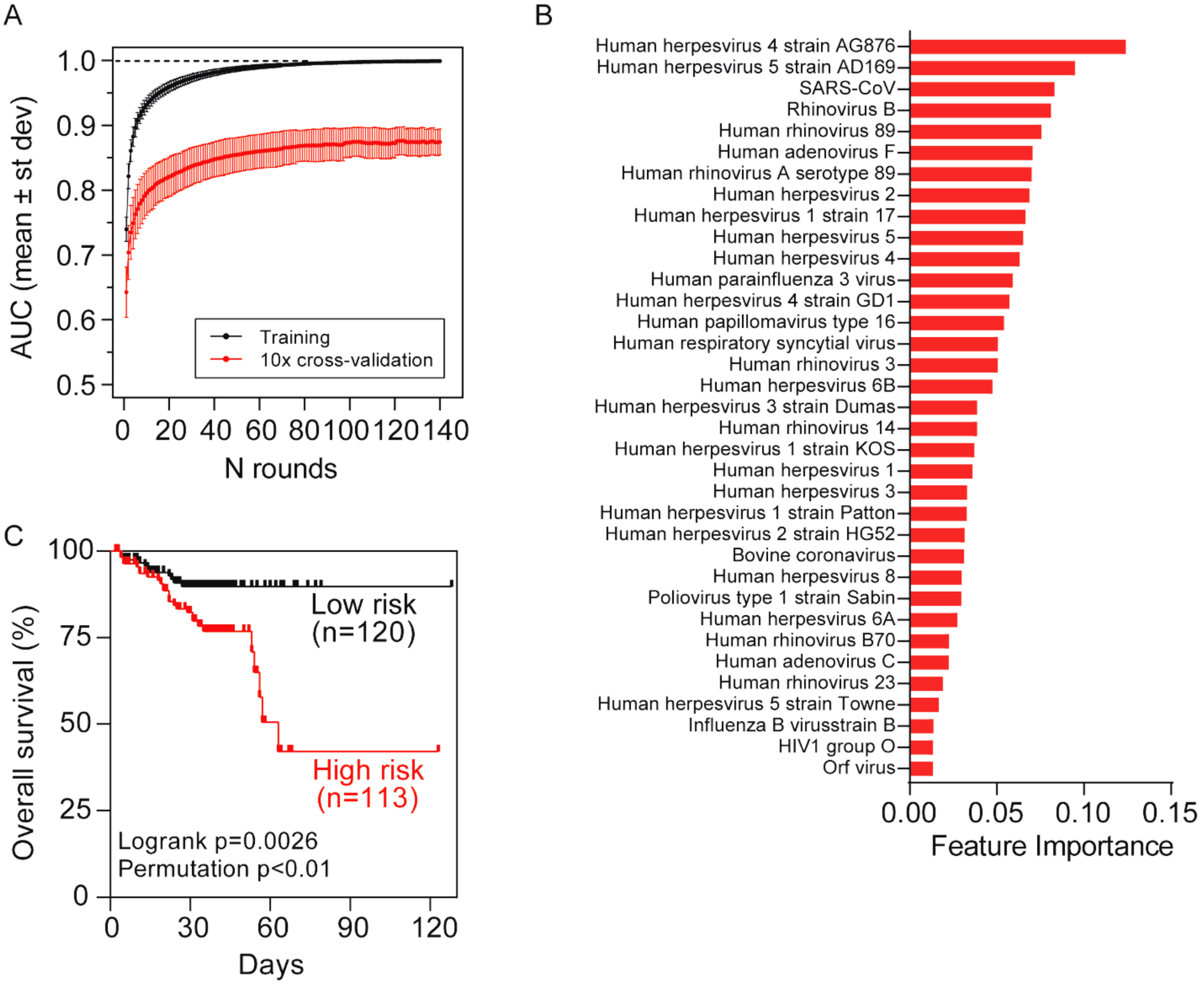

Supplemental Figure 5. Development of viral exposure signature predictive of disease severity by combining discovery and validation cohort of samples. COVID-19 positive samples from the discovery and validation cohorts were combined and balanced with ROSE for XGBoost searching for COVID-19 related virus exposure signature. (**A**) XGBoost with 10-fold cross validation for 100 iterations of balanced input data generated by ROSE. Mean AUC value of the 100 iterations with standard deviation is shown. (**B**) The resulting alternative COVID-VES consists of 35 viral strains that were selected in at least 50 iterations generated by XGBoost. (**C**) Survival risk prediction based on the 35 viral COVID-VES in low- and high-risk groups in the combined cohort. Survival time was based on days since admission. P-values were determined with Logrank and Student's t-test.
